# Characteristics Associated with the Differential Activity of Nondominant and Dominant Affected Hands in Patients with Poststroke Right Hemiparesis

**DOI:** 10.1155/2020/2387378

**Published:** 2020-05-24

**Authors:** Jen-Pei Lee, Shuya Chen, Chien-Tsung Tsai, Hsu-Chih Chung, Wen-Dien Chang

**Affiliations:** ^1^Department of Neurosurgery, Da-Chien General Hospital, Miaoli, Taiwan; ^2^Department of Physical Therapy, China Medical University, Taichung, Taiwan; ^3^Department of Rehabilitation, Da-Chien General Hospital, Miaoli, Taiwan; ^4^Department of Sport Performance, National Taiwan University of Sport, Taichung, Taiwan

## Abstract

**Objectives:**

Spontaneous arm use in patients with poststroke hemiparesis is crucial to the recovery of functional interaction. Patients with stroke and subsequent right hemiparesis have more difficulty adapting to a right-handed environment. The aim of this study was to use wearable devices to assess the asymmetry and difference in the amount of activity of the nondominant and dominant affected hands among patients with stroke and right hemiparesis. The real activity of both hands was measured to assess the correlation with various aspects of the International Classification of Functioning, Disability and Health (ICF). *Subjects and Methods*. Patients with stroke and right hemiparesis were recruited. They were divided into two groups according to the affected hand. Groups A and B comprised patients with affected nondominant and dominant hands, respectively. The Fugl-Meyer assessment-Upper Extremity (FM-UE) scores, Motor Activity Log (MAL), and hand function domain scores on the Stroke Impact Scale (SIS) were used for assessment. Patients were asked to wear smart wearable devices on both hands 24 hours a day for a month. The amount of activity in both hands was recorded and analyzed.

**Results:**

A total of 29 patients with stroke were divided into group A (*n* = 14) and group B (*n* = 15). FM-UE scores were significantly and strongly correlated with the amount of use (AOU) in the MAL. The recorded differential real activity of both hands in group B was significantly lower than that in group A. The asymmetry index of hand use was significantly less favorable in group B. However, no significant differences in AOU in the MAL, FM-UE, and hand function domain in the SIS were identified between the nondominant and dominant affected hands.

**Conclusions:**

The asymmetry and differential activity of both hands were worse in the patients with poststroke right hemiparesis, whose dominant hand was affected. However, no differences of three aspects of the ICF were found between dominant and nondominant affected hands.

## 1. Introduction

Approximately 60% of patients with stroke exhibit motor impairments of the upper extremities, which affect their social participation and quality of life [1]. The spontaneous use of hemiplegic upper extremities is a crucial stage in patients with stroke receiving rehabilitation treatments [2]. Patients with stroke could return to participating in social activities and meaningful life activities easily, if they exhibit a high degree of spontaneous affected arm use. However, spontaneous use of hemiplegic upper extremities in patients with stroke was not as expected, because of repeated unsuccessful or negative consequences of the use of paretic hand [3]. Low spontaneous paretic hand use is associated with a reduced quality of life, and the degree of spontaneous arm use affects functional interaction [4]. Based on the International Classification of Functioning, Disability and Health (ICF), the functional and activity level recovery of the upper extremities was correlated with social participation level in patients with stroke [5]. Improvements in the activity of the paretic hand and arm use could also be an indicator of the recovery of functional ability poststroke. Therefore, the body function and structure level in the ICF must be assimilated into the activity level of the upper extremities, especially spontaneous use. Integrating the amount of activity of the affected hands into real-life activities may improve quality of life, and it thus warrants investigation [6].

Recently, wearable sensors have been increasingly used to measure motor activity and asymmetries in healthy and unhealthy persons. A smart wearable device is usually a highly sensitive activity monitor that can be used to record arm movement and physical activity. A previous study used wearable devices to both record upper extremity activities and assess the asymmetry of functional hand activity in patients with stroke, and differences were observed in the manual dexterity of the less-affected hand [7]. Strong evidence supports that right-hand use is faster than left-hand use in right-handers and that the dominant hand displays higher functional performance compared with the nondominant hand [8]. In our clinical experience, asymmetry in spontaneous use of the hands and limb apraxia frequently occurred in poststroke patients, even if hand function ability was restored. Certain assessment tools, such as the Motor Activity Log (MAL) and the actual amount of use test, have been used to assess spontaneous hand or arm use. Using reliable and objective measurements of motor activity and accurately representing the amount of arm use are crucial. Technological products have been used to help therapists evaluate the motor impairment level and quantitatively propose rehabilitation programs. Some studies have employed smart wearable devices to measure the affected hand activity of patients poststroke [7, 9], because self-report questionnaires for spontaneous use of the paretic hand were not accurate in measuring hand use among patients with stroke [10]. Therefore, a smart wearable device may be a suitable measure tool for recording the activity of hand use.

A previous study focused on predictors of the amount of arm recovery following the ICF model and found that neurological response and sensorimotor function integrity in the early stroke stages were crucial predictors of long-term function recovery in the affected upper extremities [11]. Some assessment tools for patients with stroke were used to provide appropriate measure outcomes, which could alleviate the symptoms of quantification and improve occupational therapy follow-up. Measurement tools could allow therapists to adopt optimal treatment strategies, thereby helping patients with stroke to return to their previous activities of daily living [12]. For instance, the Fugl-Meyer assessment is the most commonly used assessment in patients with stroke to evaluate the body structure and function level in the ICF [13]. The MAL predominantly assesses the activity level in the ICF [13, 14]. The Stroke Impact Scale (SIS) is the tool most frequently used to assess participation level in patients poststroke [15]. The measurement of upper-limb function is crucial for optimizing occupational therapy practices [16]. Therefore, an appropriate measurement of the spontaneous use of the affected hand poststroke is crucial. However, the current activity evaluation of upper extremities often used the MAL [17], which does not specifically measure the amount of hand activity. Wearable devices have recently become increasingly common to record arm and body motion activities [18]. The devices are placed on the leg or wrist, and acceleration signals are recorded to measure the number of steps, arm movements, or physical activity [19]. Such devices could be used to measure paretic hand activity and observe spontaneous use.

The use of the dominant hand to operate everyday functional tools is often hindered in patients with stroke, and using the nondominant hand could reduce functional efficiency and increase using time. We presumed that right-handed equipment was more common in an everyday environment, and thus, patients with stroke resulting in right hemiparesis may have difficulty operating and using standard equipment. Compensating with the other hand often occurs because of temporal and spatial defects after brain hemisphere damage [20]. Some studies have demonstrated that nonparetic hand use increases 2-6 times more compared with the paretic hand in patients with poststroke hemiparesis [9, 21]. However, clinical studies comparing the differences in hand use and asymmetry of the nondominant and dominant hands among patients with stroke during real-life interactions are scarce. Transferring therapeutic gains from occupational therapy into daily activities is crucial for patients with stroke [22]. The therapeutic effects are based on the transfer package, which increases the use of the affected hand [22]. We further determined that patients with stroke and hemiparesis have difficulty achieving therapeutic gains in real-world activities requiring hand use from the transfer package, especially if the paretic hand is the dominant hand. The real amount of paretic hand activity is often unknown when patients with stroke leave the rehabilitation department to return to the community. Therefore, we used a wearable device to measure the amount of hand activity in patients with stroke and observed the differences in hand use between both upper extremities. The aim of this study was to compare the differences in the amount of activity of the nondominant and dominant affected hands among patients with poststroke right hemiparesis and further correlate the differential real activity of the use of each hand according to the various aspects in the ICF.

## 2. Methods

### 2.1. Participants

This study was an observational study. Patients with chronic hemiparesis (stroke onset > 6 months) after the first stroke event were recruited from a rehabilitation center in a hospital between 2017 and 2019. A sample size of at least 14 participants per group was estimated following a study by Molle Da Costa et al. [23]. The study process was explained to the patients, and written informed consent was obtained. This study procedure was approved by the Institutional Review Board of China Medical University Hospital (No. CRREC-106-106).

#### 2.1.1. Inclusion and Exclusion Criteria

The inclusion criteria were as follows: patients with poststroke right hemiparesis; Brunnstrom stage III proximal and distal motor functions of the affected upper limbs; no other disease affecting the performance of the upper limbs; able to walk independently without assistive devices; no cognitive impairment; and could express themselves and communicate satisfactorily. Exclusion criteria were participants that could not cooperate to wear a wearable device for 1 month.

#### 2.1.2. Demographic and Clinical Characteristics

**Figure 1:**
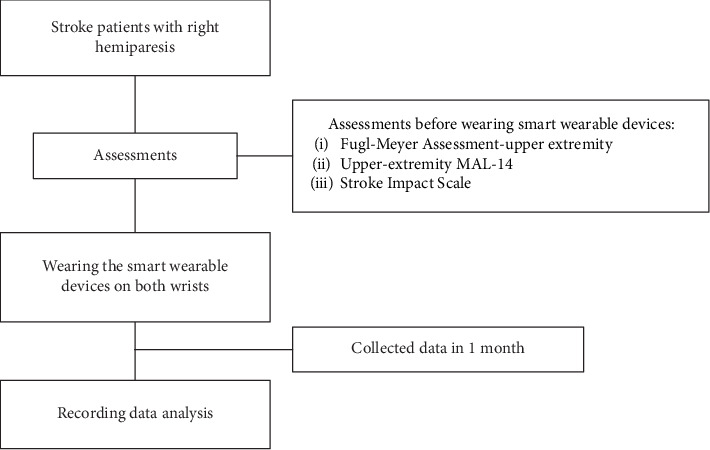
Flowchart for study procedure.

The demographic and clinical characteristics of the participants, including age, gender, dominant hand, stroke time, and stroke type, were recorded. The patients were divided into group A, if their affected hand was the nondominant hand, and into group B, if their affected hand was the dominant hand (Figure 1). The dominant hand is an individual's preferred hand to use for daily activities. The Edinburgh handedness inventory was used to assess the dominant hand in daily activities. The inventory contains 10 main items regarding the hand used for writing, drawing, throwing, using scissors, using a toothbrush, using a chopstick, using a spoon, using a broom, striking a match, and screwing a jar. The therapist asked the patients with a stroke which hand they had favored for these activities prestroke. The scores of the right and left hands were summed up, respectively. The formula ^“^(right − hand scores–left − hand scores)/(right − hand scores + left − hand scores) × 100” was used to determine which hand was dominant [24].

### 2.2. Smart Wearable Device

After completing all assessments, the patients with stroke were asked to wear a smart wearable device (Fitness Tracker Mi Band-3, Xiaomi, China) on both wrists for 24 hours a day for a month. Devices were worn on both wrists using Velcro wristbands all day, except during activities such as swimming or other activities involving contact with water. The Fitness Tracker Mi Band-3 resembles a watch and is lightweight (18.1 g) and small (25.4 cm × 2.5 cm × 76.2 cm). The supplying battery is estimated to last 20 days and do a recharge at the fifteenth day of the recording. The device was reliable for measuring upper-limb activity [25]. The output data of hand use activity were collected from the devices.

### 2.3. Assessments

Based on aspects of ICF levels, the Fugl-Meyer, MAL, and SIS scores were used to assess three components among patients with stroke. Three self-reported questionnaires were filled out by the patients and assessed by a physical therapist.

#### 2.3.1. Fugl-Meyer Assessment-Upper Extremity

Fugl-Meyer assessment is an evaluation tool for a performance-based impairment index in patients with stroke [26]. The Fugl-Meyer assessment-Upper Extremity (FM-UE) was used to assess motor recovery among patients. Each item in the FM-UE was assessed on a 3-point scale (0 = cannot perform, 1 = partially performs, and 2 = fully performs), and the total score for 66 points was calculated. The FM-UE has been designed to assess upper extremity motion and is used for patients with poststroke hemiparesis with high reliability (Cronbach's *α* > 0.80) and moderate validity (mean concurrent validity > 0.70) [27].

#### 2.3.2. Motor Activity Log

Upper-extremity MAL-14 is a subjective measure of the upper limb activity in actual daily living and belongs to the activity domain of the ICF model. MAL-14 uses a semistructured interview to review the spontaneous use of the upper extremity by using the amount of use (AOU) scale in the daily life of patients with stroke. The MAL has a high reliability (Cronbach's *α* = 0.94 ~ 0.99) and moderate to high validity (Spearman's rho = 0.64 ~ 0.99) to assess the spontaneous use of affected hands in daily life [28]. It was used in this study to determine the extent of the activities performed by patients with stroke by using the upper extremities, and the scale ranged from 0 (never used) to 5 (normal use) during 30 daily functional tasks [29]. The average score of the total AOU in MAL-14 was calculated.

#### 2.3.3. Stroke Impact Scale

The SIS (version 3.0) was used to assess the effect of hemiparesis on life interactions among patients. It is the participation level domain of the ICF model. It has high reliability (Cronbach's *α* = 0.89) and moderate validity (Spearman's rho = 0.50 ~ 0.73) to assess the functional effect of stroke [30]. Patients' ability to use their paretic hands were assessed using the five items “carry heavy objects,” “turn a doorknob,” “open a can or jar,” “tie a shoelace,” and “pick up a dime” in the hand function domain of the SIS. The item score ranged from 1 (could not do it at all) to 5 (not difficult at all), and the total score was converted to a hand function domain score, with scores ranging from 0 to 100 [31].

### 2.4. Monitor Data Extraction and Calculation

The Fitness Tracker Mi Band-3 stored hand movements using a 3-axis accelerometer. The data were extracted using Mi Band software. The hand activity count provides an index of hand activity, which was calculated across three axes and was combined using a composite vector magnitude. The average activity of the dominant and nondominant hands (counts/day) was assessed. The differential activity of the patients' hands was calculated by subtracting the activity of the nondominant hand from that of the dominant hand. A positive value of differential activity of the hands indicated higher contributions from the dominant hand compared with the nondominant hand, whereas a negative value indicated the opposite result. The asymmetry index was also calculated using the following formula: (activity of dominant hand − activity of nondominant hand)/(activity of dominant hand + activity of nondominant hand) × 100 [25]. The positive or negative asymmetry index represented more contributions from the dominant or nondominant hand, respectively.

### 2.5. Statistical Analysis

All data were analyzed using SPSS (version 20, IBM, NY, USA). Descriptive statistics were used for continuous variables, which were presented as mean ± standard deviation. For comparison between the two groups, the Mann-Whitney *U* test was used to analyze the parameters of patient age, onset of stroke, FM-UE scores, AOU in MAL, SIS scores, and activity of the hand. Fisher's exact test was used to analyze the sex and stroke type of patients. The Spearman correlation coefficient was used to analyze relationships among the FM-UE scores, AOU in MAL, SIS scores, and differential activity of the hands. The strength of the correlation was graded as *r*_s_ = 0 ~ 0.19 for very weak; *r*_s_ = 0.2 ~ 0.39 for weak; *r*_s_ = 0.4 ~ 0.59 for moderate; *r*_s_ = 0.6 ~ 0.79 for strong; and *r*_s_ = 0.8 ~ 1 for very strong relationships [32]. The significance level was set at *p* < 0.05.

## 3. Results

A total of 29 patients with poststroke right hemiparesis (age = 58.87 ± 11.83 years; weight = 81.62 ± 12.69 kg; height = 163.75 ± 12.38 cm; mean FM-UE score = 45.36 ± 11.68; mean AOU in MAL scores = 1.96 ± 1.45; mean score of hand function domain in SIS = 54.68 ± 16.12; and onset of stroke = 4.29 ± 2.54 years) were recruited for the study. All of them were diagnosed as having left cerebral hemisphere damage. As illustrated in Table 1, the patients were divided into group A (*n* = 14), and group B (*n* = 15). The groups did not differ significantly in age, sex, onset of stroke, or stroke type (*p* > 0.05). As shown in Table 2, the differential activity of the hands did not exhibit significant differences that correlated with the FM-UE scores (*r* = 0.28, *p* = 0.18), AOU in MAL (*r* = 0.09, *p* = 0.67), and SIS scores (*r* = 0.13, *p* = 0.52). A strong correlation was observed between AOU in the MAL and FM-UE (*r* = 0.71, *p* = 0.001).

**Figure 2:**
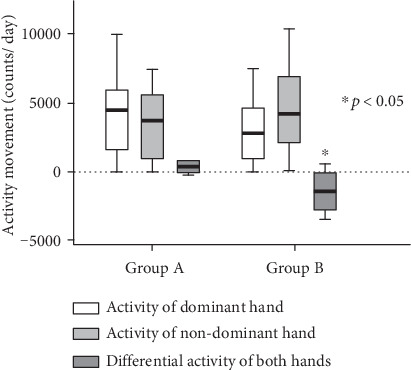
Activities of the hands between two groups.

Regarding the activity of hands, the activity of the dominant hand in group B was lower than that of the nondominant hand (Table 3). However, no significant differences were observed in the activities of the dominant and nondominant hands between the two groups. Furthermore, the differential activity of both hands in group B was significantly lower than that in group A (*p* = 0.001; Figure 2). Moreover, a significant difference in asymmetry index was observed in both groups (*p* = 0.02).

## 4. Discussion

The purpose of the present study was to compare the amount of activity of the nondominant and dominant hands and correlate the differential activity of both hands with various aspects of the ICF. We used a wearable device to monitor and record the amount of activity of both hands in patients with poststroke right hemiparesis. Our findings indicated that the AOU scores in the MAL were significantly correlated to the FM-UE scores among patients with poststroke hemiparesis. The findings further indicated that when the dominant hand was affected, the compensatory use of the nondominant hand was increased and the asymmetry index was further reduced. However, no significant differences were observed in AOU in the MAL and FM-UE scores between the nondominant and dominant affected hands.

After the onset of a stroke, the cost of rehabilitation and care of patients with a long-term disability is considerably high [33]. A cerebrovascular accident caused by brain bleeding or embolism indirectly affects the motor function of the limbs. Hemiplegia poses a major challenge to the independent life of patients with stroke after discharge and seriously affects their quality of life [34]. Clinical evidences indicate that hemiparesis of the upper extremities in patients with stroke presents with motor defects, including involuntary arm use, muscle weakness, inability to stretch and grab objects, and loss of functional activity [35]. Reducing dyskinesia and returning to daily living abilities are the basic expectations and most crucial rehabilitation goals for patients with stroke [36]. However, clinical observations and empirical findings have revealed low rates of spontaneous arm use in patients with stroke after discharge [37]. The AOU in the MAL was used to measure the affected upper extremity and detect the presence of learned nonuse. We determined that the average score of AOU in the MAL for dominant-hand-affected patients, measured using smart wearable devices, was lower than that for patients who were nondominant hand affected, but the difference was nonsignificant. Sterr et al. observed the ratio of spontaneous to forced hand use in stroke patients with hemiparesis and determined that when they were asked to use the affected hands, most of them completed required movement tasks [37]. The difference between forced and spontaneous hand use was as high as 75.8%. This phenomenon is known as “learned nonuse” and refers to the difference between motor capability and expected use of the upper extremities [38].

In this study, we recruited patients with poststroke right hemiparesis, because they would have more challenge adapting to a right-handed environment. Our findings indicated that FM-UE was significantly and strongly correlated with the AOU in MAL (*p* < 0.05). The findings indicated that the measure of body functions and structure level was related to the measure of activity level in the ICF, because both questionnaires were focused on assessing affected hand use in patients with poststroke right hemiparesis. FM-UE was used to assess the impairment severity in patients with strokes, and the MAL was used to evaluate the self-reported use of the paretic hand in daily life tasks. The paretic hand caused motor impairment affecting manual dexterity, and as a result, it affects the performance of daily activities [39]. However, no correlation was observed between the differential activity of both hands and FM-UE, MAL, or SIS scores, respectively (*p* > 0.05). We presumed that these self-reported questionnaires for paretic hand use did not accurately measure to perform the differences of hand real activity in daily life. The hand function domain in the SIS of the paretic hand was used to estimate the interaction of impairment, activity, and daily life in the community among patients with stroke [40]. The SIS questionnaire as scores of FM-UE and AOU in MAL was used to measure the functional activity of the paretic hand but did not represent the hand real activity in daily life, which resulted in no significant correlations to the differential activity of both hands. A previous study indicated that approximately 50% of patients with stroke who return to the community continue to encounter difficulties in performing daily activities, which affects their independent daily living [41]. However, when patients with stroke stopped using the affected hand for daily activities and started using the nonaffected hand, learned nonuse caused for long-term compensatory use [42]. This phenomenon created a vicious circle in motion motivation, negative feedback, spontaneous hand use, and neuroplasticity reduction, resulting in a delay in the recovery of hand function [43].

Molle Da Costa et al. indicated that patients with poststroke learned nonuse of the upper limb had lower AOU and quality of movement in the MAL, accompanying lower functional independence and quality of life compared with patients with upper-limb use [23]. Although the ability of patients with stroke recovered after rehabilitation, they tended to underestimate the spontaneous use capability of the affected hand [44]. The patients with stroke used their nondominant hand to compensate for the time and efficiency of hand-use performance. Sabaté et al. further indicated that the left cerebral hemisphere was dominant for motor planning, and a left-brain stroke could affect the movement velocity and activity in both hands [45]. It also resulted in trajectory control deficit and influenced daily living activity and functional performance [46]. Patients with stroke must rely on their nondominant hand as a primary manipulator for functional activities. Furthermore, they frequently become excessively dependent on caregivers and it adversely affects their independent capacities of activity and participation. We also found no significant differences in the FM-UE scores, AOU in MAL, and SIS scores between group A (nondominant hand affected) and group B (dominant hand affected). These results indicated no differences in function, activity, and participation levels between the nondominant and dominant affected hand, which may be because of neglect for spontaneous arm use.

This study revealed a difference in the activity of hand use between the dominant and nondominant affected hands and found a significant difference and asymmetry of activities in both hands. However, few studies have focused on the use of wearable devices to record the amount of both hands' activities among patients with poststroke hemiparesis. Rand and Eng observed the 3-day functional activity among patients with poststroke hemiparesis, measured using accelerometers on both hands and on their waist [47]. After undergoing a therapeutic exercise program, the patients' motor function improved and the number of gait steps in the lower extremities also increased. But the spontaneous movements of the upper limbs did not increase [47]. Rand and Eng further recorded spontaneous movements of the upper limbs, which were assessed using the MAL and a hand accelerometer for 1 year in patients with stroke [48]. They found that the AOU in MAL was higher than that in the movement data recorded by the hand accelerometer, because of recall bias in self-reported MAL. Some studies have indicated that accelerometers worn on the wrist provide a valid assessment to record hand use [49–51]. In a previous study on constraint-induced movement therapy, Uswatte et al. found that 3-day accelerometer recordings were strongly correlated with the quality of movement in the MAL (*r* = 0.91, *p* < 0.05), but no significant correlation was observed between accelerometer recordings and the AOU in MAL (*r* = 0.44, *p* > 0.05) [52]. In the present study, the differential activity of both hands in the smart wearable device recording and the AOU in MAL exhibited an extremely weak correlation (*r* = 0.09). Psychological cognitive factors may have caused the spontaneous use of hands reported in the MAL score to be higher than the actual amount of hand activity, particularly because the MAL required self-report of quality of movement. Therefore, the sensitivity and accuracy between the MAL and wearable device recording for hand activities in patients with stroke require further investigation.

In the present study, the included patients with stroke had a score of 45.36 ± 11.68 in the FM-UE, indicating a relatively high function. Fleming et al. demonstrated that patients with poststroke hemiparesis had moderate to high function of their upper extremity (40 < scores of FM − UE < 66) but exhibited low spontaneous use of their hands [53]. Because the motor functions of the paretic upper extremity do not considerably affect their daily life, the patients reduce the spontaneous use of hand. In our study, the quality of functional movement in all patients was assessed, and the FM-UE scores were above 40. The activities of the dominant and nondominant affected hands did not differ significantly. However, asymmetry and differential activity were observed in both hands when the stroke affected the dominant hand.

In the present study, we considered that smart wearable devices could assist occupational therapists in measuring accurate paretic hand activity in patients with stroke. This approach could provide more information for rehabilitation follow-up. Smart wearable device recording indicated that stroke patients with dominant right hands had asymmetry activities of the hand use in the activities of daily living. Notably, differential measurement results could be used to assess clinical outcomes following interventions. However, this study had some limitations. First, smart wearable devices worn on both wrists recorded movement activity, but the movement recorded by these devices cannot represent an accurate amount of hand activity. Second, this study only measured hand activity among patients with poststroke right hemiparesis. Further studies in patients with poststroke left hemiparesis and comparison of the differences are warranted. Third, the small sample size reduced the statistical power of our study and the lack of long-term recording made it difficult to analyze follow-up changes in spontaneous paretic hand use.

## 5. Conclusions

Patients with poststroke right hemiparesis face more challenges in adapting to a right-handed environment, which affects spontaneous paretic hand use. Our results indicated that the FM-UE score was significantly and strongly correlated with the AOU in MAL. Compared with the dominant and nondominant affected hand, the asymmetry and differential activity of both hands were significantly different in the stroke patients, whose dominant hand was affected. However, no differences in FM-UE, AOU in the MAL, and hand functional domain in SIS scores were observed between the dominant and nondominant affected hands among patients with poststroke right hemiparesis.

## Figures and Tables

**Table 1:** Demographic data of the stroke patients.

Items	Group A (*n* = 14)	Group B (*n* = 15)
Age^a^	59.21 ± 12.65	58.80 ± 11.24
Gender (male/female)	10/4	10/5
Onset of stroke (years)^a^	4.14 ± 2.44	4.50 ± 2.79
Stroke type (hemorrhagic/infarction stroke)	5/9	4/11

^a^Presented as mean ± standard deviation.

**Table 2:** The correlations between FM-UE, AOU of MAL, SIS, and differential activity of both hands.

Items	Differential activity of both hands	FM-UE	AOU of MAL
FM-UE	0.28	—	—
AOU of MAL	0.09	0.71^∗^	—
SIS	0.13	0.30	0.12

∗*p <* 0.05; *FM*-UE: Fugl-Meyer assessment-Upper Extremity; AOU: amount of use; MAL: Motor Activity Log; SIS: Stroke Impact Scale.

**Table 3:** Comparisons of FM-UE, MAL, SIS, and hand activity in both groups.

Items	Group A (*n* = 14)	Group B (*n* = 15)
FM-UE	48.35 ± 6.30	42.40 ± 13.78
AOU of MAL	2.24 ± 1.33	1.71 ± 1.47
SIS	55.35 ± 17.70	54.00 ± 13.49
Activity of dominant hand (counts/day)	4126.13 ± 2914.02	3053.76 ± 2611.75
Activity of nondominant hand (counts/day)	3365.19 ± 2463.88	4504.74 ± 3201.03
Differential activity of both hands (counts/day)	760.94 ± 1163.20	−1450.98 ± 1399.73^∗^
Asymmetry index	4.21 ± 36.07	−30.47 ± 31.72^∗^

∗*p <* 0.05; FM-UE: Fugl-Meyer assessment-Upper Extremity; AOU: amount of use; MAL: Motor Activity Log; SIS: Stroke Impact Scale.

## Data Availability

The data used to support the findings of this study are included within the article.
